# Adrenalectomy Improves Blood Pressure and Metabolic Control in Patients With Possible Autonomous Cortisol Secretion: Results of a RCT

**DOI:** 10.3389/fendo.2022.898084

**Published:** 2022-06-02

**Authors:** Valentina Morelli, Sofia Frigerio, Carmen Aresta, Elena Passeri, Flavia Pugliese, Massimilano Copetti, Anna Maria Barbieri, Silvia Fustinoni, Elisa Polledri, Sabrina Corbetta, Maura Arosio, Alfredo Scillitani, Iacopo Chiodini

**Affiliations:** ^1^Unit for Bone Metabolism Diseases and Diabetes, Istituto Auxologico Italiano, Istituto di Ricovero e Cura a Carattere Scientifico (IRCCS), Milan, Italy; ^2^Unit of Endocrinology, Fondazione Istituto di Ricovero e Cura a Carattere Scientifico (IRCCS) Cà Granda-Ospedale Maggiore Policlinico, Milan, Italy; ^3^Department of Clinical Sciences and Community Health, University of Milan, Milan, Italy; ^4^Endocrinology and Diabetology Service, Istituto di Ricovero e Cura a Carattere Scientifico (IRCCS) Istituto Ortopedico Galeazzi, Milan, Italy; ^5^Unità Operativa di Endocrinologia Fondazione Istituto di Ricovero e Cura a Carattere Scientifico (IRCCS)—”Casa Sollievo della Sofferenza”—Hospital, San Giovanni Rotondo, Foggia, Italy; ^6^Unit of Biostatistics Fondazione Istituto di Ricovero e Cura a Carattere Scientifico (IRCCS)—”Casa Sollievo della Sofferenza”—Hospital, San Giovanni Rotondo, Foggia, Italy; ^7^Lab Toxicology, Fondazione Istituto di Ricovero e Cura a Carattere Scientifico (IRCCS) Cà Granda-Ospedale Maggiore Policlinico, Milan, Italy; ^8^Department of Biomedical, Surgical and Dental Sciences, University of Milan, Milan, Italy; ^9^Department of Medical Biotechnology and Translational Medicine, University of Milan, Milan, Italy

**Keywords:** adrenal incidentaloma, hypercortisolism, hypertension, diabetes, adrenalectomy

## Abstract

**Objective:**

The best approach to patients with adrenal incidentaloma (AI) and possible autonomous cortisol secretion (PACS) is debated. The aim of this study was to assess the metabolic effect of adrenalectomy in AI patients with PACS in relation to cortisol secretion parameters, peripheral activation, and glucocorticoid sensitivity.

**Design:**

This is a multicenter randomized study (NCT number: NCT04860180).

**Methods:**

Sixty-two AI outpatients (40–75 years) with AI >1 cm and cortisol after overnight dexamethasone suppression test (F-1mgDST) between 50 and 138 nmol/L were randomized to adrenalectomy (Arm A) or a conservative approach (Arm B). Fifty-five patients completed the 6-month follow-up, 25 patients in Arm A (17 female patients, aged 62.5 ± 10.4 years) and 30 patients in Arm B (24 female patients, 66.1 ± 9.1 years). Plasma adrenocorticotroph hormone (ACTH), 24-h urinary free cortisol, 24-h urinary free cortisone, F-1mgDST, glucose, lipids, glycated hemoglobin (HbA1c) levels, blood pressure (BP), body weight, and treatment variations were assessed. The 24-h urinary free cortisol/cortisone ratio (an 11-beta hydroxysteroid dehydrogenase type 2 activity marker), BclI, and the N363S variants of glucocorticoid receptor (GR) polymorphisms were also evaluated.

**Results:**

BP control improved in 68% and 13% of the subjects in Arm A and Arm B, respectively (*p* = 0.001), and the glycometabolic control improved in 28% and 3.3% of the subjects in Arm A and Arm B patients, respectively (*p* = 0.02). Arm A subjects more rarely showed the BP and/or glycometabolic control worsening than Arm B patients (12% and 40%, respectively, *p* = 0.03). The surgical approach was independently associated with BP amelioration (OR 3.0, 95% CI 3.8–108.3, *p* < 0.001) but not with age, F-1mgDST levels, BMI, and hypertension and diabetes mellitus presence at baseline. The 24-h urinary free cortisol/cortisone ratio and the presence of sensitizing GR polymorphisms were not associated with the surgical outcome. The receiver operating characteristic (ROC) curve analysis showed that the BP control amelioration was associated with F-1mgDST [area under the curve (AUC), 0.82 ± 0.09 *p* = 0.012]. The F-1mgDST cutoff with the best compromise in predicting the BP amelioration was set at 75 nmol/L (sensitivity 77%, specificity 75%).

**Conclusions:**

AI patients with PACS benefit from surgery in terms of BP and glycometabolic control.

## Introduction

The condition of possible autonomous cortisol secretion (PACS) in patients with incidentally discovered adrenal masses [adrenal incidentaloma (AI)] has been recently defined as a mild biochemical cortisol excess without the clinical features specific to hypercortisolism (such as striae rubrae, dorsocervical fat pad, easy bruising, hypertricosis, and moon facies). The European Society of Endocrinology (ESE) guidelines, released in 2016, reserve the PACS definition to AI patients with cortisol after 1-mg overnight dexamethasone suppression test (F-1mgDST) between 50 nmol/L and 138 nmol/L (1). The PACS condition has recently become a topic of growing interest, since it has been consistently suggested to be associated with chronic consequences, particularly with diabetes mellitus (DM), hypertension (HT), and an increased mortality risk (2–8). In addition, at variance with the clinically full-blown form of hypercortisolism [Cushing’s syndrome (CS)], which is considered a rare disease, PACS has an estimated prevalence of 0.8%–2% in the adult population. Indeed, PACS has been described to occur in 5%–30% of subjects with AI, which, in turn, are found in up to 7% of subjects above 60 years of age (5, 8).

The PACS diagnosis is still a debated issue, as no test or combination of tests have been demonstrated to be fully reliable in diagnosing this subtle cortisol excess (2, 9–11). Overall, most authors agree on the fact that F-1mgDST is the best parameter to diagnose PACS, as the cortisol levels after the overnight administration of 1 mg dexamethasone is associated with the possible cortisol-related comorbidities such as HT, DM, and osteoporosis (5, 12, 13). At variance, urinary free cortisol (UFF) and midnight salivary cortisol determinations have not been proved to reliably identify PACS patients (14, 15). Besides the cortisol secretion, peripheral cortisol activation and glucocorticoid sensitivity (cortisol milieu) play a relevant role in determining the clinical and biochemical presentation of these patients (16).

Notwithstanding these uncertainties regarding the PACS diagnosis, most studies show that the recovery from PACS could lead to the amelioration of DM, HT, and possibly obesity (OB) (17). However, the vast majority of the available data come from retrospective studies with a wide variability in the criteria used for defining PACS, and the only available prospective study (18) has been carried out on a population of patients with a degree of cortisol hypersecretion higher (i.e., F-1mgDST levels above 70 nmol/L, mean F-1mgDST levels 166 nmol/L) than that considered by the ESE guidelines for the PACS definition (i.e., F-1mgDST levels between 50 and 138 nmol/L). Importantly, nowadays, in PACS patients, surgery is rarely recommended, i.e., only in patients with possible cortisol-related comorbidities (1). From the ESE guidelines release onwards, no randomized prospective studies have been carried out. Therefore, whether or not surgery is beneficial in PACS patients is still unknown.

In this study, we report data from a randomized clinical trial designed to assess the effect of adrenalectomy in AI patients with PACS. The complete study design and endpoints are available at ClinicalTrials.gov website (NCT number: NCT04860180). In this paper, we report data on the modifications at 6 months of body weight (BW), blood pressure (BP), glucose control, and lipid metabolism (primary outcomes of the study). The secondary outcome of the study was to evaluate the role of cortisol milieu in predicting these changes, if present.

## Patients and Methods

### Patients

The study has been approved by the Ethical Committee of Milan Area B (n. 809_2015, resolution n. 516 25/03/2016). In September 2016, the study protocol was started by our research group to assess the effect of adrenalectomy in AI patients with PACS. A cohort of consecutive PACS patients have been randomized to surgery or to a conservative approach in order to evaluate the effect of adrenalectomy on several outcomes possibly associated with hypercortisolism, including variations of BW, BP, and glycometabolic control. The study has been conducted at three different Italian hospitals: Fondazione IRCCS Ca' Granda, Milan, IRCCS Istituto Ortopedico Galeazzi, Milan, and IRCCS “Casa Sollievo della Sofferenza” Hospital, San Giovanni Rotondo, Foggia. The enrollment ended in February 2020.

The inclusion criteria were as follows: (i) age between 40 and 75 years, and (ii) diagnosis by imaging of unilateral AI larger than 1 cm with radiological features at computed tomography consistent with an adrenocortical adenoma (homogeneous and hypodense, Hounsfield units <10 or with proven radiological dimensional stability). The exclusion criteria were as follows: (i) hypogonadism, thyrotoxicosis, chronic renal failure and hepatic disease, alcoholism, rheumatologic and hematological disease, and eating disorders, including binge eating disorder, bulimia nervosa, and anorexia nervosa; (ii) intake of drugs influencing cortisol and dexamethasone metabolism or cortisol secretion; (iii) signs or symptoms specific to hypercortisolism (striae rubrae, dorsocervical fat pad, easy bruising, hypertricosis, and moon facies); (iv) possible metastatic diseases or radiologic appearance not consistent with an adrenocortical adenoma; (v) biochemical evidence of pheochromocytoma and aldosteronoma; (vi) ACTH dependency; and (vii) incomplete diagnostic work-up. Patients with AI larger than 5 cm have been excluded, as surgery is mandatory in these subjects (19).

**Figure 1:**
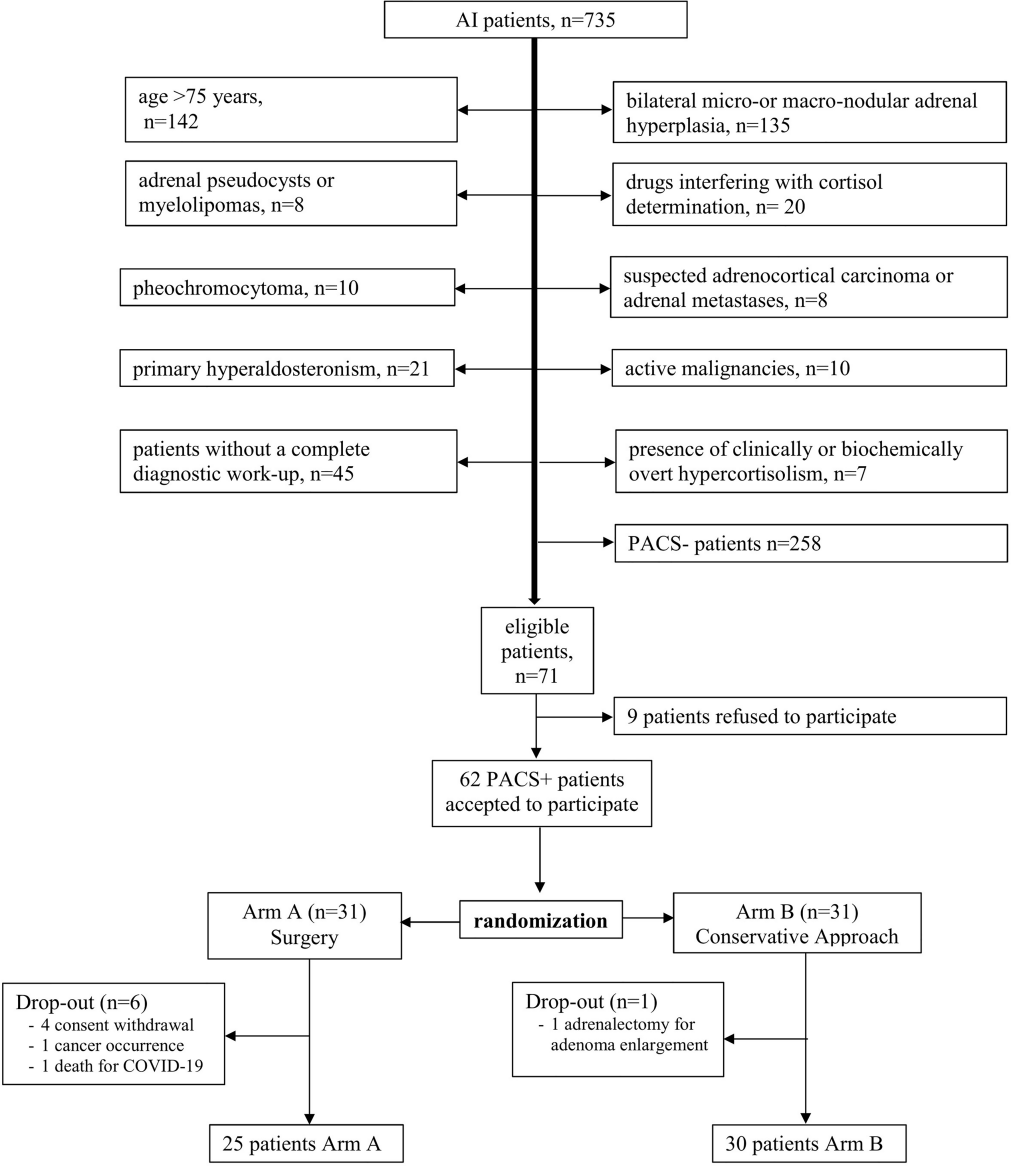
Enrollment procedure. AI, adrenal incidentaloma; PACS, possible autonomous cortisol secretion; PACS+, patients with possible autonomous cortisol secretion; PACS-, patients without possible autonomous cortisol secretion.

The enrollment procedure along with the number of the included and excluded patients and the reasons for their exclusion is depicted in **Figure 1**. A total of 735 patients with AI have been evaluated for inclusion. Three hundred ninety-nine patients have been excluded on the basis of the above-mentioned inclusion and exclusion criteria. The remaining 336 patients underwent the F-1mgDST level determination. Patients with F-1mgDST below 50 nmol/L (*n* = 258) have been excluded, as they have not been affected by PACS. All patients with confirmed (at least twice) F-1mgDST above 138 nmol/L (*n* = 7) have been excluded as they have been affected by biochemically overt hypercortisolism (1). All patients with confirmed F-1mgDST levels between 50 and 138 nmol/L were eligible for study inclusion.

On the basis of the inclusion and exclusion criteria, the study has been proposed to 71 patients, of whom 9 refused to participate. Therefore, 62 patients were enrolled: 31 were randomized to surgical intervention (Arm A) and 31 were randomized to a conservative approach (Arm B).

A block randomization was used to reduce bias and achieve balance in the allocation of participants to treatment arms.

In Arm A, four patients withdrew consent before surgery, one patient dropped out due to the occurrence of breast cancer after baseline assessments, and one patient died of coronavirus disease 2019 (COVID-19) before surgery; in Arm B, one patient underwent adrenalectomy for a rapid adenoma size increase due to internal nodule bleeding (after 5 months’ follow-up). Eventually, 55 patients (25 in Arm A and 30 in Arm B) completed the study protocol.

Antihypertensive treatment is specified in **Table 1**. Regarding antidiabetic drugs, 3 out of 5 diabetic patients of Arm A were treated with metformin (mean dose 1,666 ± 1258 vs. 1,333 ± 764 mg at baseline and follow-up, respectively); 2 patients did not take any drug. Among 6 diabetic patients of Arm B, 4 patients were treated with metformin at baseline (mean dose 1,475 ± 769 mg/day), 5 patients were treated at follow-up (mean dose 1,175 ± 495 mg/day), and 1 patient was started with 100 mg of DPPIV inhibitor after baseline evaluation.

**Table 2:** Clinical and biochemical modifications in PACS patients conservatively treated and surgically treated and at baseline and at 6 months’ follow-up.

	Baseline			6-month follow-up		
	Arm A (*n* = 25)	Arm B (*n* = 30)	*p*	Arm A (*n* = 25)	Arm B (*n* = 30)	*p*
**Age** (years)	62.5 ± 10.4 (41–75)	66.1 ± 9.1 (41–75)	0.18	63.2 ± 10.3 (41–76)	66.5 ± 9.1 (42–76)	0.21
**Gender** (Female)	17 (68)	24 (80)	0.36	17 (68)	22 (80)	0.36
**BMI** (kg/m^2^)	27.4 ± 5.3 (18.6–44.0)	26.8 ± 5.2 (18.7–37.4)	0.67	27.2 ± 5.0 (18.3–39.4)	26.4 ± 5.1 (18.1–38.1)	0.59
**Diameter of adenoma** (cm)	3.1 ± 0.8 (1.5–4.5)	2.8 ± 0.7 (1.4–4.3)	0.22	–	–	–
**ACTH** (pg/ml)	8.3 ± 2.7 (5.0–19.9)	9.8 ± 4.3 (5.0–13.5)	0.16	26.0 ± 10.6 (10.8–57.0)	11.6 ± 4.9 (5.0–24.2)	**<0.0001**
**F- 1mgDST** (nmol/L)	88.3 ± 24.8 (52.4–124.2)	80 ± 22.1 (50–132.5)	0.19	19.3 ± 5.5 (13.8–30.3)	85.6 ± 27.6 (52.4–138)	**<0.0001**
**UFF** (nmol/24 h)	72.0 ± 32.8 (29–140.8)	59.3 ± 30.1 (19.9–118.7)	0.14	48.8 ± 27.6 (19.3–113.2)	60.2 ± 32.3 (19.9–136.9)	0.22
**UFF/UFE ratio**	0.27 ± 0.10 (0.1–0.45)	0.26 ± 0.17 (0.1–0.9)	0.96	0.22 ± 0.10 (0.1–0.4)	0.26 ± 0.15 (0.1–0.7)	0.61
**Patients with obesity (%)**	8 (26.7)	8 (32)	0.45	6 (24.0)	7 (23.3)	1.00
**Body weight change (%) (stable/worse/better)**	–	–	–	19/2/4 (76/8/16)	26/1/3 (86.7/3.3/10)	0.48
**Patients with DL (%)**	15 (60)	21 (70)	0.33	15 (60)	20 (66.7)	0.41
**DL control (%) (stable/worse/better)**	–	–		23/1/1 (92/4/4)	23/4/3 (76.7/13.3/10)	0.31
**SBP (mmHg)**	139.1 ± 14.0 (100–170)	139.7 ± 15.3 (110–165)	0.88	133.2 ± 12.8 (105–160)	142.9 ± 16.7 (110–170)	**0.02**
**DBP (mmHg)**	82.2 ± 10.1 (70–100)	77.7 ± 10.2 (60–97)	0.12	77.1 ± 8.9 (60–90)	78 ± 10.7 (60–100)	0.74
**Patients with HT (%)**	16 (64)	22 (73)	0.75	14 (56)	22 (73)	0.14
**HT grade**	1.0 ± 0.8 (0–3)	0.9 ± 0.7 (0–2)	0.62	0.6 ± 0.5 (0–2)	1.1 ± 0.8 (0–2)	0.02
**Number of antihypertensive drugs**	2 (2)	1 (2)	0.69	1 (2)	1.5 (2)	0.46
**BP control (%)** **(stable/worse/better)**	**–**	**–**	**–**	7/1/17 (28/4/68)	18/8/4 (60/26.7/13.4)	**0.001**
**Glycated hemoglobin (mmol/mol)**	40.8 ± 6.6 (32–57)	39.5 ± 7.1 (30–67.0)	0.50	39.6 ± 5.4 (31.0–51.0)	39.8 ± 6.6 (27–57)	0.91
**Patients with DM (%)**	5 (20)	6 (20)	1.00	5 (20)	6 (20)	1.00
**Patients with IGT/IFG (%)**	7 (28)	9 (30)	1.00	5 (20)	10 (33.3)	0.37
**DM grade**	1 ± 1.4 (0–4)	0.9 ± 1.1 (0–4)	0.84	0.8 ± 1.2 (0–3)	1.1 ± 1.2 (0–4)	0.34
**GL control (%) (stable/worse/better)**	**–**	**–**	**–**	16/2/7 (64/8/28)	23/6/1 (76.7/20/3.3)	**0.03**

Data are mean ± SD with range in parenthesis or absolute number with percentage in parenthesis, or as median with interquartile range in parenthesis for the number of antihypertensive drugs. BMI, body mass index; F-1mgDST serum cortisol levels after 1-mg dexamethasone suppression test; UFF, urinary free cortisol (normal values 3–43 μg/24 h); ACTH, adrenocorticotroph hormone (normal values 10–55 pg/ml). IFG, impaired fasting glucose; IGT, impaired glucose tolerance. PACS was diagnosed in the presence of F-1mgDST above 50 nmol/L and below 138 nmol/L. BP, blood pressure; SBP, systolic BP; DBP, diastolic BP; HT, hypertension; GL, glycometabolic; DL, dyslipidemia.

In bold: p<0.05.

Informed consent was obtained from each patient after explanation of the purpose and nature of all procedures used.

### Methods

In all patients, at baseline and at 6 months, the following variables have been assessed: plasma adrenocorticotroph hormone (ACTH), UFF, 24-h urinary free cortisone (UFE) and F-1mgDST levels, BP, body mass index (BMI), lipid metabolism, fasting glucose, insulin, glucose after 75-mg oral glucose tolerance test (OGTT), and glycated hemoglobin (HbA1c) levels. In Arm A, the 6-month evaluations started after the substitutive corticosteroid therapy withdrawal.

The blood venous samples were taken after 10-h fasting in the morning, and all samples were stored at −20°C until analysis.

Plasma ACTH levels at 8:00 a.m. were measured by chemiluminescence (Immulite 2000, Siemens Medical Solutions Diagnostics, Los Angeles, California, normal values 10–55 pg/ml). Serum cortisol concentrations were measured by the second-generation monoclonal immunoassay Elecsys Cortisol II with a limit of detection of 1.5 nmol/L, a limit of quantitation of 2.0 nmol/L, an interassay CV ranging from 1.9% to 10.1%, and an intra-assay CV from 1.5% to 5.4% (Elecsys Cortisol Immunoassay, Roche Diagnostics, Mannheim, Germany, on CobaS E 602). The determination of UFC (reference range up to 43 μg/24 h) and UFE (reference range up to 122 μg/24 h) was performed by liquid chromatography–tandem mass spectrometry (LC-MS/MS, coefficient of variation <10%, accuracy between 98% and 100%, and limit of quantification 1 µg/L, as previously described for both determinations) (20). As per our protocols, the 24-h urine collection has been repeated in case of possible inadequacy 11-beta hydroxysteroid dehydrogenase type 2 activity marker (i.e., reduced 24-h urine quantity and/or creatinine excretion). The 24-h urinary UFF/UFE ratio was used as a marker of 11beta-hydroxysteroid dehydrogenase type 2 (HSD11B2) activity—the enzyme inactivating cortisol to its inactive form cortisone (the higher the R-UFF/UFE ratio, the lower the HSD11B2 activity).

BP was measured following the European Society of Cardiology/European Society of Hypertension (ESH/ESC) guidelines: the patients sat for several minutes in a quiet room before beginning BP measurements; at least two measurements spaced by 1–2 min were taken, and additional measurements were taken if the first two were quite different; a standard bladder was used (12–13 cm long and 35 cm wide), but a larger and a smaller bladder were available for fat and thin arms, respectively; the cuff was placed at the heart level, whatever the position of the patient; at first visit, BP was measured in both arms to detect possible differences due to peripheral vascular disease. In this instance, the higher value was taken as the reference one and all the subsequent measurements were done at the same arm. HT was defined as the detection of systolic BP (SBP) and/or diastolic BP (DBP) above 140 mm Hg and 90 mm Hg, respectively, and/or the need for any antihypertensive treatment (21). DM was diagnosed using the American Diabetes Association current clinical practice recommendations (22). HOMA-IR Index was calculated through the following formula: blood glucose (mmol) × insulin (UI/ml) ÷ 22.5. Dyslipidemia was defined as serum triglyceride levels of at least 150 mg/dl or high-density lipoprotein cholesterol levels of less than 40 mg/dl in men and 50 mg/dl in women. Patients were also considered dyslipidemic if any antidyslipidemic treatment was given (23).

After baseline evaluations, patients of Arm A were addressed to surgery. In Arm B, patients with borderline-elevated BP or grade 1 hypertension, with prediabetes, or with overweight were suggested to follow intensive lifestyle behavior changes, while patients with grade 2 to 3 hypertension, with not fully controlled diabetes (HbA1c >53 mmol/mol), with obesity, or with dyslipidemia were addressed to cardiologists and/or diabetologists in order to consider therapy modifications. The medical treatment was not standardized but personalized to obtain the best possible result in individual patients (21).

Improvement or worsening during follow-up has been defined as follows: (i) for BW, in the presence of a >5% BW decrease or increase (24), respectively; (ii) for BP, if the non-hypertensive patients passed from a pre-hypertension category to another or if the hypertensive patients passed from a hypertension grade to another (21) or if the dosage and/or number of antihypertensive drugs was at least halved or doubled, respectively; (iii) for glycometabolic control, if glucose levels passed from a category to another, following the ADA criteria, or if the dosage of antidiabetic drugs was at least halved or doubled, respectively, or if an antidiabetic drug was withdrawn or an additional antidiabetic drug was needed, respectively, or if HbA1c passed from grade 4 (>53 mmol/mol) to grade 3 (53–48 mmol/mol) or to grade 2 (48–42 mmol/mol) or to grade 1 (42–39 mmol/mol) or to grade 0 (<39 mmol/mol) (22); (iv) cholesterol levels were considered improved or worsened if they passed from a category to another in agreement with the Adult Treatment Panel III criteria (24).

Genotypes for BclI and the N363S variants of GR gene were assessed as previously described (25). As homozygous BclI and heterozygous N363S polymorphisms have been associated with increased cortisol sensitivity, we decided to classify patients with homozygous BclI and/or heterozygous N363S polymorphism as carriers of the sensitive variant of GR (25).

### Sample Size and Statistical Analysis

On the basis of the results found in terms of BP and glycometabolic control, the sample size evaluated in the present study consented to have a power of 0.8 (type I error 0.05).

Statistical analysis was performed by SPSS version 26.0 statistical package (IBM SPSS, Milan, Italy). The normality of data distribution was tested by Kolmogorov–Smirnov test. Results are expressed as mean ± standard deviation and with range for normally distributed continuous variables or as absolute frequencies and percentages for categorical variables. The comparisons of continuous variables between Arm A and Arm B at baseline and after 6-month follow-up have been performed by one-way ANOVA and Bonferroni correction for *post-hoc* analysis. The categorical variables have been compared by using *χ*^2^ test or Fisher exact test, as appropriate.

The amelioration or worsening of BP and glycometabolic control has been used as a primary and secondary endpoint, respectively, and their prevalence was compared between Arm A patients and Arm B patients. The receiver operating characteristic (ROC) curve assessed the association between the endpoints and the baseline ACTH, UFC, UFF/UFE ratio, and F-1mgDST levels.

The logistic regression analysis was used to assess the independent association between the amelioration or worsening of BP or glycometabolic control and the surgical treatment or conservative approach after adjusting for possible confounding variables, such as age, gender, BMI, presence of DM and HT, and F-1mgDST levels at baseline. *p*-values <0.05 were considered statistically significant. The paired-samples *t*-test was used to compare baseline and 6-month HOMA-IR Index values.

## Results

All Arm A patients underwent laparoscopic adrenalectomy without complications. All patients were treated with corticosteroid substitutive therapy for the first 2 months, and then adrenal function was regularly evaluated by corticotropin test. We had a 40% prevalence of adrenal insufficiency. Adrenal function was evaluated every 3 months until normalization. The mean duration of impaired adrenal function was 12.3 ± 9.0 months (6–35). Patients with hypoadrenalism showed baseline F-1mgDST levels comparable to patients without (3.7 ± 0.7 vs. 3.0 ± 1, *p* = 0.107).

The comparisons of the characteristics of Arm A and Arm B patients between baseline and at 6-month follow-up are reported in **Table 2**. In Arm A, the 6-month evaluations started after the HPA axis recovery and substitutive corticosteroid therapy withdrawal.

**Table 1:** Number of patients taking a specific class of antihypertensive treatment.

	Arm A		Arm B	
	Baseline (*n* = 16)	End of Follow-up (*n* = 14)	Baseline (*n* = 22)	End of Follow-up (*n* = 22)
**Any antihypertensive agent *n* (%)**	16 (100)	13 (93)	22 (100)	22 (100)
**ACE inhibitor or angiotensin-receptor blockers *n* (%)**	13 (81.3)	9 (64.3)	15 (68.2)	16 (72.7)
**Beta-blocker *n* (%)**	5 (16.1)	4 (12.9)	11 (35.5)	11 (35.5)
**Any diuretic, *n* (%)**	5 (17.9)	5 (17.9)	9 (32.1)	9 (32.1)
**Calcium antagonists *n* (%)**	10 (26.3)	5 (13.2)	12 (31.6)	11 (28.9)

p > 0.5 for all comparisons¸ ACE, angiotensin-converting-enzyme.

At baseline, Arm A and Arm B patients were comparable in terms of age, gender distribution, BMI, prevalence of obesity, HT, DM, impaired fasting glucose (IFG), and impaired glucose tolerance (IGT). Mean SBP, DBP, and HbA1c levels; AI size; ACTH; UFF; UFF/UFE ratio; and F-1mgDST levels were not different between Arm A and Arm B patients. Duration of hypertension and diabetes was comparable between the two groups (hypertension: 11.5 ± 10.2 and 9.4 ± 7.7 years in Arm A and Arm B, respectively, *p* = 0.551; diabetes: 3.8 ± 3.9 and 4.6 ± 6.5 years in Arm A and Arm B, respectively, *p* = 0.825).

A sensitive variant of the GR gene was found in 6 patients equally distributed among the two groups (*p* = 0.674).

As expected, at 6 months’ follow-up, Arm A patients showed higher ACTH and lower UFF and F-1mgDST levels than Arm B patients, while age, BMI, prevalence of obesity, HT, DM, IFG, and IGT were comparable between the 2 groups.

**Figure 2:**
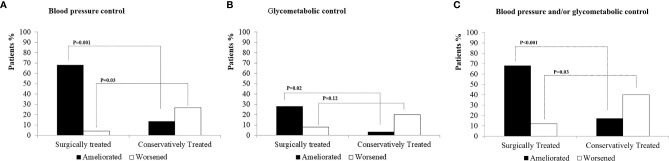
Amelioration and worsening of blood pressure and/or glycometabolic control in surgically treated or conservatively managed AI patients with PACS. AI, adrenal incidentaloma; PACS, possible autonomous cortisol secretion; PACS+, patients with possible autonomous cortisol secretion; PACS-, patients without possible autonomous cortisol secretion. **(A)** Amelioration and worsening of blood pressure control. **(B)** Amelioration and worsening of glycometabolic control. **(C)** Amelioration and worsening of blood pressure and/or glycometabolic control.

Overall, the BP variations at 6 months were associated with the surgical treatment or conservative approach. Indeed, BP control improved in 68% of Arm A subjects and in 13.4% of Arm B subjects (*p* = 0.001), while it worsened in 26.7% of Arm B subjects and in 4% of Arm A subjects (*p* = 0.03) (**Figure 2A**). At 6-month follow-up, mean SBP levels were lower in Arm A than in Arm B subjects. In the whole sample, the logistic regression analysis showed that the surgical approach was independently associated with the improvement of BP control (odds ratio 3.0, 95% confidence interval 3.8–108.3, *p* < 0.001) but not with age, BMI, F-1mgDST levels, HT, and DM presence at baseline (**Table 3**). The ROC curve showed that the BP control amelioration was not associated with the UFF and ACTH levels, while it was associated with F-1mgDST [area under the curve (AUC), 0.82 ± 0.09 *p* = 0.012]. At variance, the BP control worsening was not associated with F-1mgDST, ACTH, and UFF levels. The F-1mgDST cutoff with the best compromise in predicting the BP amelioration was set at 75 nmol/L (sensitivity 77%, specificity 75%).

**Table 3:** Independent association of the blood pressure control improvement with the assignment to a surgical or conservative approach in PACS patients after adjusting for possible confounding factors.

	OR	95% CI	*p*-value
**Surgical approach (yes)**	3.0	3.8–108.3	**<0.001**
**Age (1-year increase)**	1.47	1.1–8.5	0.12
**BMI (kg/m^2^ increase)**	1.05	0.90–1.25	0.54
**F-1mgDST (27.6 nmol/L increase)**	1.36	0.60–3.10	0.46
**Hypertension (yes/no)**	1.2	0.20–6.21	0.90
**Diabetes (yes/no)**	2.34	0.33–16.52	0.40

OR, odds ratio; 95% CI, 95% confidence interval, BMI, body mass index; PACS, possible autonomous cortisol secretion.

In bold: p<0.05.

The glycometabolic control variations showed a similar behavior. Indeed, the glycometabolic control improved in 28% and 3.3% of Arm A patients and Arm B patients, respectively (*p* = 0.02), while it worsened in 8% and 20% of Arm A patients and Arm B patients, respectively (*p* = 0.12) (**Figure 2B**). In both arms, glucose control did not worsen among diabetic patients with scarce glycometabolic control (i.e., with HbA1c >53 mmol/mol) but mainly among euglycemic subjects, who developed an IFG/IGT status, or among diabetic patients with good glycometabolic control (i.e., with HbA1c <53 mmol/mol), in whom HbA1c worsened. The insulin resistance, as measured by the HOMA-IR Index, remained stable in Arm A (baseline: 2.7 ± 2.6, end of follow-up: 2.6 ± 1.9, *p* = 0.84), but significantly increased in Arm B patients over time (baseline: 2.5 ± 0.8, end of follow-up: 4.9 ± 9.4, *p* = 0.025). The ROC curve showed that the amelioration or worsening of glucose metabolism control was not associated with F-1mgDST, UFF, UFF/UFE ratio, and ACTH levels.

As shown in **Figure 2C**, the amelioration of BP and/or glycometabolic control was more frequent in Arm A than in Arm B patients (68% vs. 17%, respectively, *p* < 0.001), while these latter patients more frequently showed worsening of BP and/or glycometabolic control (40% vs. 12%, *p* = 0.03). No relationship was found between the adrenal insufficiency occurrence and the BP and/or glycometabolic control amelioration (data not shown).

Comparing baseline UFF/UFE ratio levels between patients with and those without amelioration of BP and/or glycometabolic control, we did not find any statistically significant difference (0.26 ± 0.1 vs. 0.27 ± 0.2, *p* = 0.827, respectively). Moreover, in Arm A, baseline UFF/UFE ratio was 0.28 ± 0.1 in patients with amelioration of BP and/or glycometabolic control and 0.24 ± 0.1 in patients without (*p* = 0.454), while in Arm B, baseline UFF/UFE ratio was 0.20 ± 0.1 in patients with amelioration of BP and/or glycometabolic control vs. 0.28 ± 0.2 in patients without (*p* = 0.395). Similarly, the prevalence of a sensitive variant of GR gene was comparable between patients with and those without amelioration of BP and/or glycometabolic control (11.1% vs. 13.8%, *p* = 0.582, respectively).

## Discussion

The present prospective randomized study was aimed at evaluating the effect of adrenalectomy as compared with a conservative approach on BP and glycometabolic control in AI patients with PACS. The secondary outcome of the study was to evaluate the role of cortisol secretion, peripheral activation, and glucocorticoid sensitivity in predicting possible changes.

We found that adrenalectomy more frequently ameliorated BP and glycometabolic control than the conservative approach, while the latter was associated with a more frequent worsening of BP and insulin resistance.

To date, the best approach in AI patients with PACS is a matter of debate, with some authors suggesting surgery in the presence of at least 2 complications of cortisol excess (26), while others, including the ESE guidelines, suggest a more prudent approach (27, 28). Indeed, a conservative approach could be preferable in PACS patients without possible cortisol-related comorbidities (29), even considering that tumor growth is clinically modest in these patients (30). These discordances are due to the lack of homogeneity among the studies that have assessed the effect of the surgical approach in patients with PACS. Indeed, the criteria for defining the condition of PACS have been notably different among the different studies, as some authors included patients with clear autonomous cortisol secretion (i.e., with F-1mgDST above 138 nmol/L), while others evaluated patients with a less severe cortisol hypersecretion (5, 31). This lack of homogeneity explains why, notwithstanding a meta-analysis that suggested the possible beneficial effect of surgery in patients with PACS (17), the ESE guidelines remained extremely prudent on advising the surgical treatment in PACS patients. This position is also justified by the fact that the vast majority of data are derived from retrospective analyses, and that the only available prospective randomized trial (18) has been performed in patients with a degree of cortisol secretion higher (i.e., F-1mgDST levels above 70 nmol/L, mean F-1mgDST levels 166 nmol/L) than that expected in PACS patients following the ESE guidelines (i.e., F-1mgDST between 50 and 138 nmol/L). Thus, although that study suggested a beneficial effect of surgery in patients with mild hypercortisolism, its results could not be considered meaningful for patients with PACS, as defined by the ESE guidelines.

To the best of our knowledge, this is the first prospective randomized study faced with the problem of how to manage AI patients with PACS as defined by the ESE guidelines. The present results deserve interest as AI patients with PACS represent a consistent part (up to 30%) of all AI patients. Indeed, the finding of a beneficial metabolic effect of surgery in AI patients with PACS and, on the contrary, of a possible negative effect of a conservative approach, if confirmed by other authors and in a longer follow-up period, may support the need for a revision of the current recommendations for these patients. Interestingly, we found that the amelioration of BP control was independent of the presence of HT and DM at baseline and also of the degree of cortisol secretion as mirrored by the F-1mgDST levels. This finding, again, is important in the light of the current ESE recommendations, which suggest considering surgery in the presence of comorbidities possibly associated with cortisol excess. Moreover, F-1mgDST levels were predictive of the amelioration of BP control after surgery, and interestingly, the F-1mgDST cutoff with the best diagnostic accuracy in predicting the improvement of BP control was set at 75 nmol/L. This cutoff could become a useful threshold for considering surgery in conservatively managed AI patients with PACS, also considering that in PACS patients not eligible for surgery, specific medical treatment for hypercortisolism is being developed (32). On the other hand, this finding also supports the idea that, regardless of the presence of cortisol excess-related symptoms, the F-1mgDST cutoff of 138 nmol/L that suggests surgery in AI patients should be reconsidered. Indeed, the available data show that the incidence of cardiovascular complications is increased in patients with F-1mgDST above 50 nmol/L (2–4, 7, 31), and recently, even lower F-1mgDST levels have been suggested to define non-functioning AI (9). In addition, as a close relationship between UFF/UFEE ratio and hypertension, in both menopausal women and CS, has been described in previous studies (16, 33), we hypothesized the role of HSD11B2 activity (evaluated by the UFF/UFE ratio) in determining surgical outcomes. Actually, we did not find any difference in 11BHSD2 activity between patients with and without BP or glycometabolic improvement, probably because, at variance with CS, in mild hypercortisolism, a full enzyme saturation is not achieved (34). However, larger studies are needed to assess the possible use of the UFF/UFE ratio as a predictor of the response to therapy.

This study has some limitations. First, we did not perform the ambulatory BP monitoring for the evaluation of BP control, and we did not standardize medical treatments among patients of Arm B. However, all patients with unsatisfactory BP control were evaluated by cardiologists in order to consider therapy modifications. Second, the worsening of glucose control in the conservative group (Arm B) could suggest that a therapeutic inertia can exist, thus representing a study bias. However, even though such a bias could not be completely excluded, we believe that it is improbable anyway. Indeed, in both arms, glucose control did not worsen among diabetic patients with scarce glycometabolic control but mainly among euglycemic subjects, who developed an IFG/IGT status, or among diabetic patients with good glycometabolic control, in whom HbA1c worsened. Accordingly, the degree of insulin resistance worsened in the conservatively treated patients. Third, we are aware that the different number of dropout patients between the two groups may have affected results. However, the fact that the minimum sample required for the study to be adequately powered has been reached anyway renders the probability of a meaningful bias due to this disparity less likely. Fourth, the evaluation of blood dexamethasone levels, a new diagnostic tool able to increase the diagnostic accuracy of F-1mgDST for suspected hypercortisolism, was not performed (35). However, F-1mgDST values were confirmed twice and cortisol was measured with a new generation assay, which is also a competitive immunoassay, but employs a different antibody (monoclonal rather than polyclonal), and it is calibrated on gas chromatography–tandem mass spectrometry (36).

Moreover, the low number of patient carriers of GR gene variants made it very difficult to find any possible associations between GR polymorphisms and the response to the therapy. Finally, the absence of specific signs and symptoms of cortisol excess in patients with PACS prevented us to estimate the duration of cortisol excess exposure, which may have played a role in influencing the effect of therapy on BP and glycometabolic control.

Notwithstanding these limitations, this prospective randomized study shows for the first time that AI patients with PACS may benefit from the mild hypercortisolism resolution in terms of BP and glycometabolic control, regardless of the presence of HT and DM at baseline. These data, if confirmed, should push us to reconsider the currently recommended approach in AI patients with PACS.

## Data Availability Statement

The original contributions presented in the study are included in the article/supplementary materials, further inquiries can be directed to the corresponding author.

## Ethics Statement

The studies involving human participants were reviewed and approved by the Ethical Committee of Milan Area B (n. 809_2015, resolution n. 516 25/03/2016). The patients/participants provided their written informed consent to participate in this study.

## Author Contributions

VM: data acquisition, analysis, and drafting of the manuscript. SoF, CA, EPa, and FP: data acquisition and analysis and interpretation of the data. AMB, SiF, EPo: data acquisition and analysis. MC: study design and data analysis. IC: study concept and design. SC, MA, AS, and IC: critical revision of the manuscript for important intellectual content, and study supervision. All authors contributed to the article and approved the submitted version.

## Funding

This study has been supported by the Grant RF 2013-02356606 from the Italian Ministry of Health to IC, VM, AS, and SC.

## Conflict of Interest

IC and CA are investigators in studies on relacorilant (Corcept Therapeutics) in patients with hypercortisolism.

IC received consulting fees from Corcept Therapeutics and HRA Pharma.

The remaining authors declare that the research was conducted in the absence of any commercial or financial relationships that could be construed as a potential conflict of interest.

## Publisher’s Note

All claims expressed in this article are solely those of the authors and do not necessarily represent those of their affiliated organizations, or those of the publisher, the editors and the reviewers. Any product that may be evaluated in this article, or claim that may be made by its manufacturer, is not guaranteed or endorsed by the publisher.
